# Novel PLGA-encapsulated-nanopiperine promotes synergistic interaction of p53/PARP-1/Hsp90 axis to combat ALX-induced-hyperglycemia

**DOI:** 10.1038/s41598-024-60208-1

**Published:** 2024-04-25

**Authors:** Rishita Dey, Sudatta Dey, Priyanka Sow, Arnob Chakrovorty, Banani Bhattacharjee, Sisir Nandi, Asmita Samadder

**Affiliations:** 1 Cytogenetics and Molecular Biology Laboratory, Department of Zoology, University of Kalyani, Kalyani, Nadia, 741235 India; 2 Department of Pharmaceutical Chemistry, Global Institute of Pharmaceutical Education and Research (Affiliated to Veer Madho Singh Bhandari Uttarakhand Technical University), Kashipur, 244713 India

**Keywords:** Computational biology and bioinformatics, Nanomedicine, Diabetes

## Abstract

The present study predicts the molecular targets and druglike properties of the phyto-compound piperine (PIP) by in silico studies including molecular docking simulation, druglikeness prediction and ADME analysis for prospective therapeutic benefits against diabetic complications. PIP was encapsulated in biodegradable polymer poly-lactide-co-glycolide (PLGA) to form nanopiperine (NPIP) and their physico-chemical properties were characterized by AFM and DLS. ∼ 30 nm sized NPIP showed 86.68% encapsulation efficiency and − 6 mV zeta potential, demonstrated great interactive stability and binding with CT-DNA displaying upsurge in molar ellipticity during CD spectroscopy. NPIP lowered glucose levels in peripheral circulation by > 65 mg/dL compared to disease model and improved glucose influx in alloxan-induced in vivo and in vitro diabetes models concerted with 3-folds decrease in ROS production, ROS-induced DNA damage and 27.24% decrease in nuclear condensation. The 25% increase in % cell viability and inhibition in chromosome aberration justified the initiation of p53 and PARP DNA repairing protein expression and maintenance of Hsp90. Thus, the experimental study corroborated well with in silico predictions of modulating the p53/PARP-1/Hsp90 axis, with predicted dock score value of − 8.72, − 8.57, − 8.76 kcal/mol respectively, validated docking-based preventive approaches for unravelling the intricacies of molecular signalling and nano-drug efficacy as therapeutics for diabetics.

## Introduction

Diabetes is a chronic, metabolic silent malady that demands comprehensive scientific management and a holistic therapeutic outlook. The leading cause of global illness burden is India's high rate of diabetes. Diabetes ranks amongst the top ten causes of death apart from cancer, respiratory disorders, and cardiovascular disease. It is considered the biggest worldwide health catastrophe of our century. This high rate of diabetes epidemic is a result of the swift socio-economic transformation, which has affected dietary habits, lifestyle choices, economic affluences, and urbanization^1^. As per the diabetic prevalence reports of the International Diabetes Federation, the global rise in diabetics was approximately 537 million individuals between the age group of 20 and 79 (representing 10.5% of all adult population within this age range). From this overview, it is predicted that a total of 643 million and 783 million individuals will have diabetes by 2030 and 2045, respectively^2^. As a result, it has been projected that although the global population will expand by 20%, the proportion of people with diabetes is expected to rise by 46%. An estimated 77 million people in India are living with diabetes and an additional 25 million are prediabetic, indicating they are at an increased risk of developing the condition shortly^3^. At least one in eleven Indians over the age of 18 are thought to be afflicted with diabetes, making India the second-highest affected country in the world behind China. According to the reports of the National Urban Diabetes study, there is a significant incidence of diabetes in three districts in West Bengal, in India estimating 13.2% in Howrah, 12% in Kolkata, and 8.7% in Burdwan^4^. Therefore, diabetics are inclined to popular synthetic anti-diabetic medications like exenatide, metformin, glimepiride, pioglitazone, repaglinide, etc. that are frequently employed in combination therapy, however, impose adverse effects that can potentially exacerbate associated health issues^5^. In this context, phytochemicals/ phyto-products have been reported to possess remarkable ameliorative facilities, cost-effectiveness, decreased cytotoxic effects, easy affordability and availability, and minimum/ least side effects making them advantageous for drug designing and discovery^6–10^.

Researchers are currently inclined towards the selective design and formulation of drugs that are not only target-specific but also lower in cost. Nanotechnology is an arena that has been exploited by millions to quench their desired quest for establishing a novel nano-mediated target-specific drug delivery tools in biological systems suitable against myriads of diseases including diabetes. The minute-sized nano-ranged particles (nanoparticles) of about 1–100 nm^11^, result in drug safety as it amplifies drug dissolving power, stability, sustainable release, and improves drug activity by preventing enzymatic degradation, improving bioavailability, and biocompatibility^12^. Added to the advantages of nanoparticles, recent studies are inclined to investigate whether the phyto-product generated nanoparticles are beneficial to mankind in a much superior manner to combat health hazards.

Molecular docking is a novel simulation to determine the energy of interaction between a small molecule and a large macromolecular protein target^13^. This interaction is expressed in terms of hydrogen bonds, van der Waals forces, hydrophobic interaction, electrostatic interaction, π-π stacking, and salt bond formations which give a score. This score is a quantitative assessment of the affinity of the ligand towards the target. The ligand with a minimal dock score may produce maximal affinity and response while interacting with the selected target. In this study, this has been used as a high throughput screening (HTS) tool for molecular simulation involving the molecular docking method^14,15^. The different software tools required for the complete docking process, from the preparation of ligand and protein to visualization of the docking interpretation are ChemDraw Ultra 8.0, Chem3D Ultra 8.0, ArgusLab 4.0.1, and Discovery Studio 3.5 (Dassault Systèmes BIOVIA, Discovery Studio 3.5, San Diego: Dassault Systèmes, 2016)^16^. Keeping pace with the time and cost of drug procurement, the present study aims to synthesize antidiabetic drugs using phytoproduct piperine (PIP) as a core ingredient, validate effective binding interaction with the proteins, and formulate nanoparticles embedded in nano-capsule using an FDA (Food and Drug Administration) approved biodegradable, environmentally friendly, and non-toxic polymeric material poly-lactide-co-glycolide (PLGA). Piperine (PIP), the bioactive component of black pepper (*Piper nigrum*), was selected due to its antioxidative^17^, anticancerous^18^, anti-inflammatory^19,20^, neuroprotective^21–23^, and hepatoprotective efficacies^24,25^. Therefore, PIP was anticipated to target different proteins entailed for anti-diabetic activities which were first predicted utilizing computer-aided molecular docking simulation. Thus, identifying bio-targets of PIP would aid in understanding the binding affinity of the ligand (drug) within the active binding cleft of the protein. This would thereby allow the precise identification of the associated signalling pathways involved in the process of combating the disease when checked in an experimental animal model to examine the rationale and routes of the drug molecule's activity.

Thus, the main objectives of the present work are: (1) to compute the binding affinity of piperine (PIP) towards the target of interest to understand ligand–protein interaction for regulating disease emergence and virulence by in silico modeling, (2) to predict the druglikeness nature of in silico screened phytocompound which determines the efficacy of the drug in the living system, (3) to effectively synthesize and characterize PLGA loaded nanopiperine (NPIP), (4) to ascertain the biodistribution, and blood–brain barrier-crossing potential of NPIP, (5) to evaluate the protective efficacy of NPIP in ALX-induced diabetes in vivo and hyperglycemic stress in vitro, (6) to recognize the biological drug targets and signalling mechanisms involved in restriction of the disease in both the models.

## Results

### Characterization of biosynthesized PLGA-encapsulated nanopiperine (NPIP)

**Figure 1:**
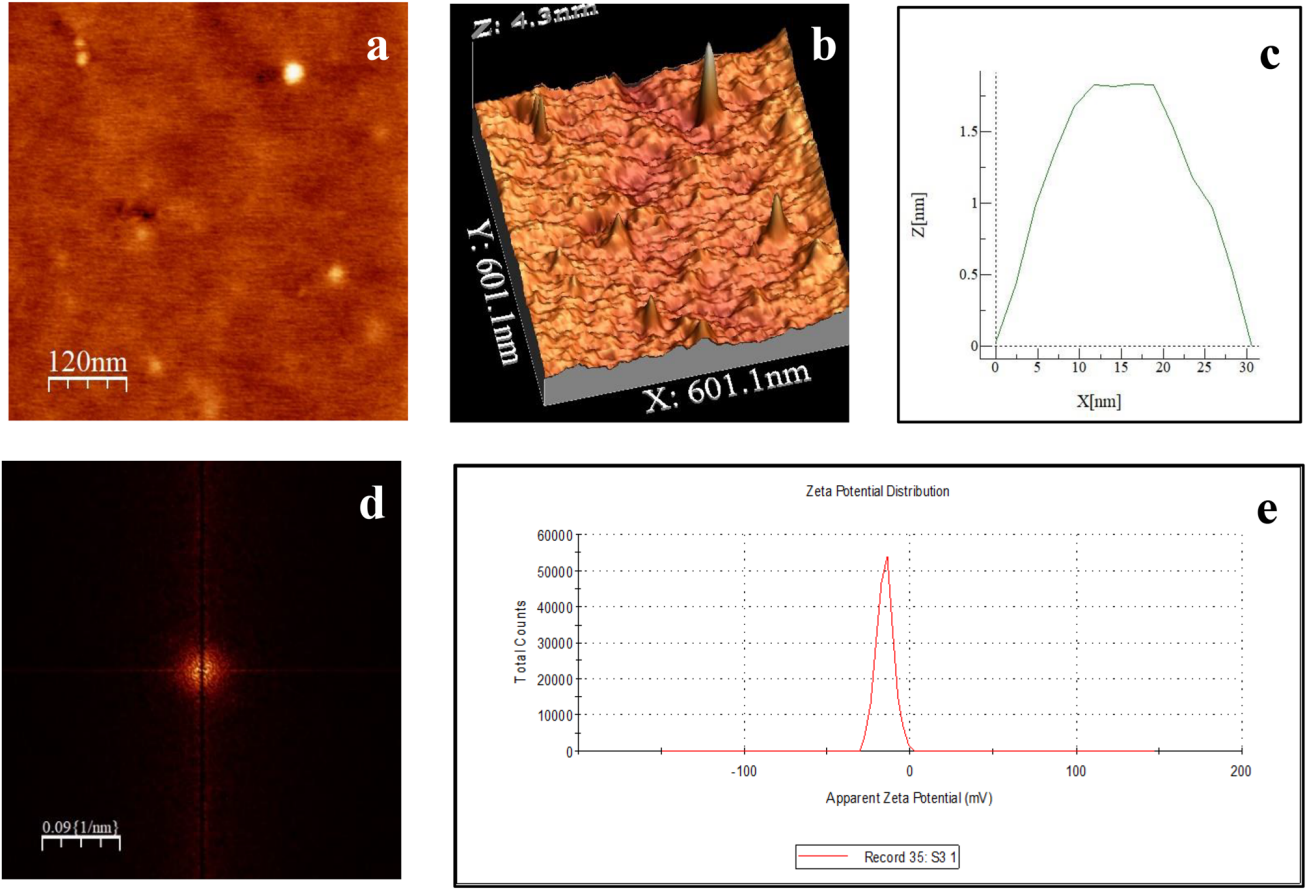
Characterization of piperine nanoparticle (NPIP): (**a**) AFM 2D image of nanoform; (**b**) AFM 3D image of nanoform; (**c**) Height profile (30 nm); (**d**) FFT spectra; (**e**) Zeta potential.

Atomic Force Microscopy (AFM) and Zeta potential were used to extensively characterize the biosynthesized PLGA-encapsulated NPIP. The AFM analysis displayed the oval form of the NPIP, and the subsequent 2D and 3D views of the AFM images demonstrated the mean dimension of the nanoparticles to be 30 ± 0.05 nm, having a smooth surface devoid of any perforations, holes, or cracks (Fig. 1a–c). A uniformness in the frequency of formation of identical nanoparticles of NPIP was evident from the 2D perspective of the Fast Fourier Transformation (FFT) analysis of the AFM data (Fig. 1d). The stability of the synthesized nanoparticles was confirmed by the zeta potential measurement, which was found to be − 6 mV (Fig. 1e). The polydispersity index (PDI) of 0.265 also established the formation of a homogenous solution of NPIP.

### Assessment of %Encapsulation Efficiency (EE%) of PIP within NPIP

The %encapsulation efficiency (EE%) of PIP inside the PLGA polymeric core of NPIP was calculated to be 86.68% from the graphical plot of free drug in different time intervals during the formulation process (Suppl. Fig. 2).

### In silico docking analysis of PIP with respective proteins

**Figure 2:**
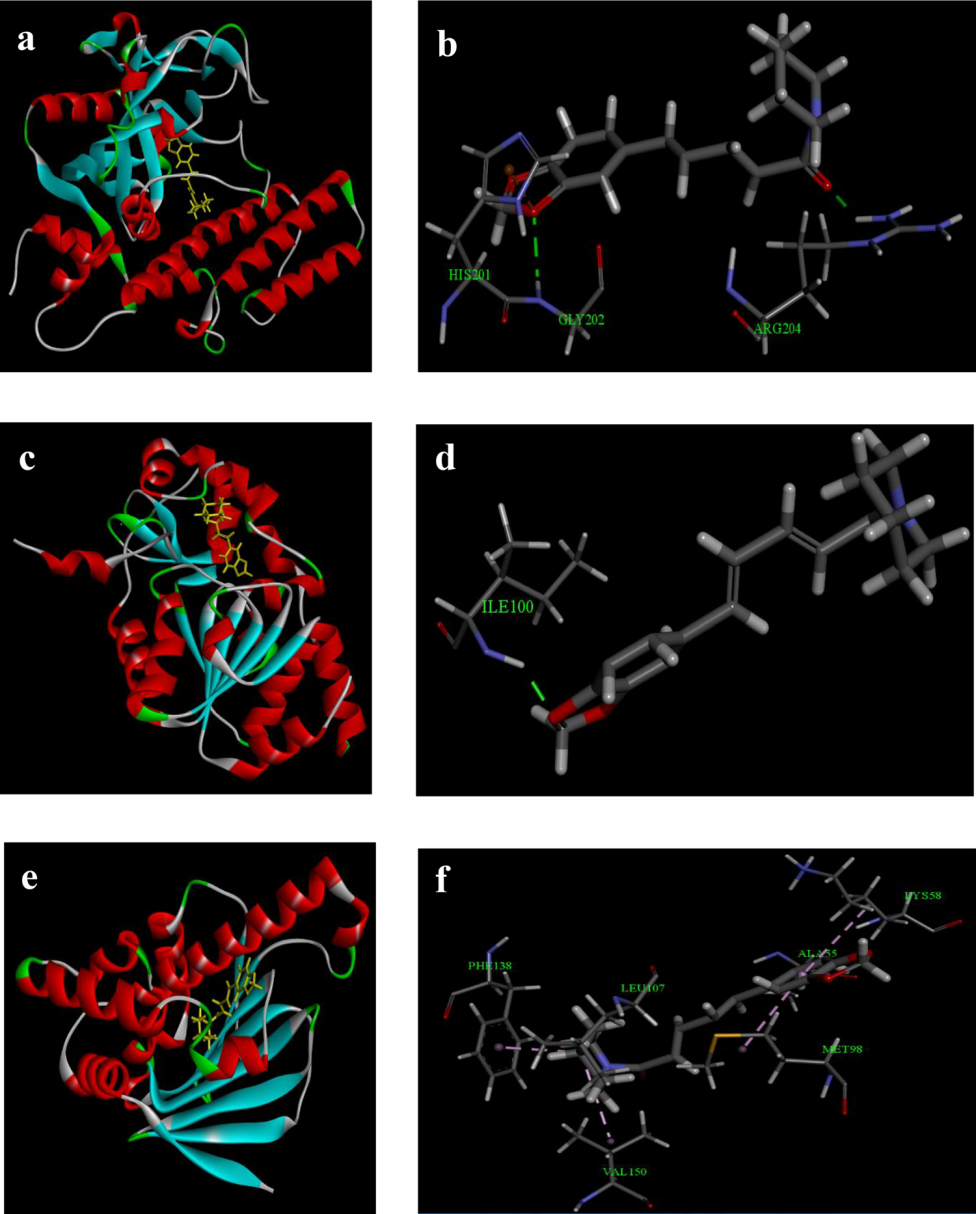
Analysis of in silico molecular docking study of piperine: (**a**) PIP and PARP-1 interaction; (**b**) Best docked conformation showing important amino acid residues of PIP and PARP-1; (**c**) PIP and p53 interaction; (**d**) Best docked conformation showing important amino acid residues of PIP and p53; (**e**) PIP and Hsp90 interaction; (**f**) Best docked conformation showing important amino acid residues of PIP and Hsp90.

Docking of the optimized protein–ligand complex permits flexible rotation of ligands within the protein’s active cleft for the generation of several conformations. The complex conformation with minimum potential energy was selected which represents the best pose of the protein–ligand binding. Docking studies of PARP-1 and PIP having a score of – 8.57 kcal/mol revealed that the carbonyl group of the ligand PIP forms hydrogen bonding interaction with the ARG204 residue of PARP-1. The docking also predicted that the methoxy moiety produces H-bonding interaction as well as forms Pi-charge electrostatic interconnection by the residues GLY202 and HIS201 respectively (Fig. 2a,b). Docking interpretation of p53 and PIP having a docking score of – 8.72 kcal/mol revealed that the methoxy group of the 1,3-dioxolane present in PIP was susceptible to hydrogen bonding interaction by the amino acid residue of ILE100. This analysis validated that methoxy moiety is the major group and has major functionality in interlinking the ligand within the active pocket of the protein to produce biological action as confirmed by the good binding score (Fig. 2c,d). The docking analysis of the Hsp90 and PIP complex displayed numerous hydrophobic interactions linking the methylene and benzene group of the ligand to the protein with a docking score of – 8.76 kcal/mol. The methylene moiety was cleft by LEU107 and VAL150 via alkyl hydrophobic interactions. The benzyl group was stacked with alkyl hydrophobic interactions by the residues ALA55, LYS58, MET98, and ILE96. PHE138 also clefts the methylene moiety via mixed pi/alkyl hydrophobic interactions (Fig. 2e,f).

### Estimation of druglike properties of PIP

**Figure 3:**
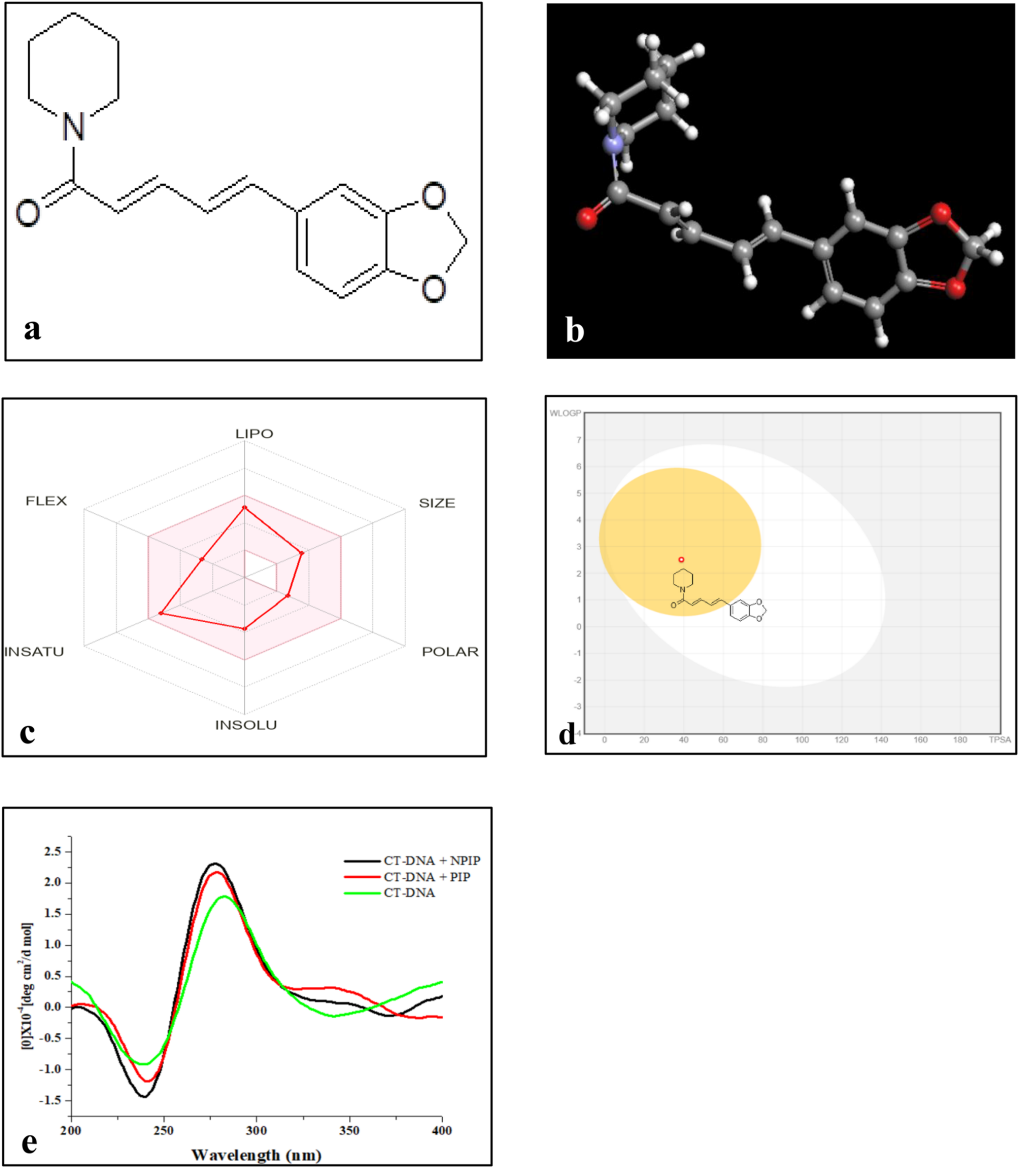
Structure of piperine and its druglikeness property: (**a**) 2D structure of piperine; (**b**) 3D structure of piperine; (**c**) Biological radar showing the suitable region of PIP; (**d**) Egan’s Boiled Egg model showing the position of PIP (white: GI tract, yellow: BBB); (**e**) Analysis of binding affinity of NPIP with CT-DNA by CD spectroscopy.

An indicator of drug-likeness is the balance of molecular properties and structural characteristics within a molecule that is vital to determine whether a specific molecule is druglike or non-druglike. The table shows the druglikeness properties of the phytocompound PIP (Suppl. Table 1) and the biological radar as obtained from the software SwissADME (Fig. 3c). According to the figure, it could be assumed that all the features fall within the range of Lipinski’s rule of 5, such as lower molecular mass, suitable XLogP value, appropriate hydrogen bond donor and acceptor, thereby establishing PIP likely to be a drug-like candidate which further serves as a rationale behind selecting this phytocompound. All these factors and arrays of characteristics mirrored the efficacy of nutraceuticals in penetrating the biological membrane without hindrance.

### ADME property prediction (absorption, distribution, metabolism, and excretion)

Caco-2-Permeability, a human colon epithelial cancer cell line representing the human intestinal absorption model, was examined to assess the absorption property of PIP, and it was shown to be within the ideal range of − 4.805 Log unit. Egan BOILED-Egg (Brain Or IntestinaL EstimateD permeation) method-based prediction using the SwissADME tool also validated the rate of absorption of PIP in the gastrointestinal tract (white zone) as well as its permeability in penetrating the blood–brain barrier (yellow zone) (Fig. 3d). Using SwissADME software, PIP was found to function as a substrate and inhibitor of the two major metabolic enzymes (Cytochrome P450) such as CYP2D6 and CYP3A4, establishing that PIP neither impede the enzymes' ability to metabolize other drugs, whose accumulation might have detrimental toxic effects nor does it lessen the beneficial effects of drugs that metabolize quickly. The excretion level of 10.873 mL/min/kg of PIP determined its efficient removal from the body following its action. All these parameters suggested that PIP might have drug-like qualities to be employed in addressing illness (represented in Suppl. Table 2). These parameters were calculated and checked via software analysis to establish compliance with their standard ranges.

### Analysis of the interaction of NPIP with CT-DNA by circular dichroism study (CD)

The CD spectroscopy of the phytocomponent with double strands of DNA provided significant information regarding the chemical interaction between nanoparticles and nucleotides in terms of peak shifting. The CT-DNA CD spectrum showed the expected characteristics of a B-form DNA, including a positive CD band at 273 nm due to DNA base stacking and a negative CD band on the opposite side at 245 nm driven by an alteration in helicity. In accordance with the peak shift, after the introduction of PIP and NPIP to CT-DNA separately, the CT-DNA showed an upsurge in CD molar ellipticity following its interaction with PIP and NPIP. The degree of intensity of the negative peaks and positive peaks in the PIP-DNA and NPIP-DNA systems increased in comparison to the peaks in the CD range for free DNA (Fig. 3e). However, there was no further shift of peak observed.

### Glucose lowering effect of NPIP

**Figure 4:**
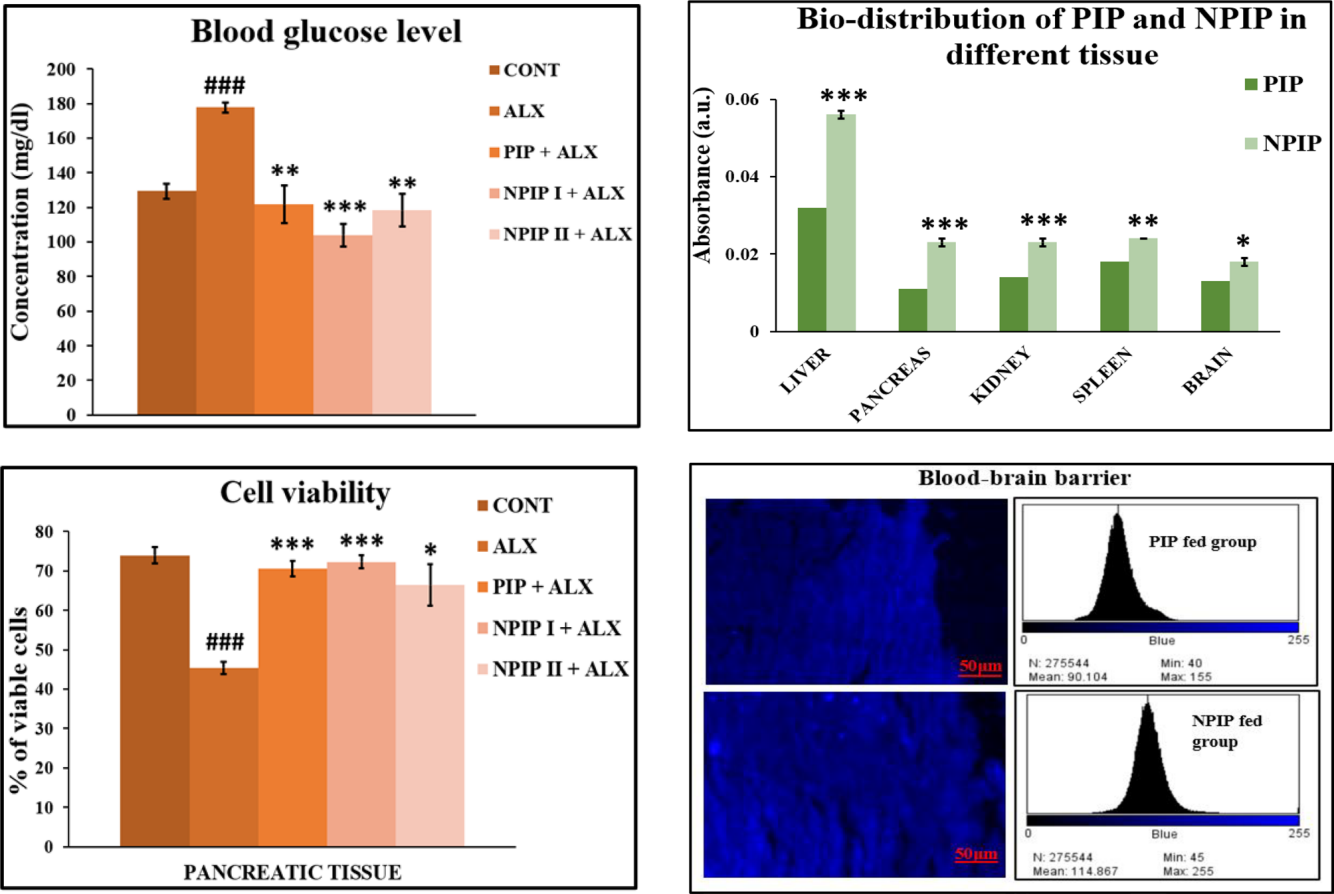
Biological parameters studied in vivo: Graphical representation showing the blood glucose level, bio-distribution of PIP and NPIP in different tissues, % cell viability in pancreatic tissue, and fluorescent imaging of brain tissue of PIP and NPIP fed mice group. ^*###*^*p* < 0.001 versus CONT, ***p < 0.001 versus ALX, **p < 0.01 versus ALX, *p < 0.05 versus ALX were considered.

When compared with the control group, alloxan treated mice group had considerably higher blood glucose levels, while pre-treated drug groups (PIP + ALX, NPIP I + ALX, NPIP II + ALX) showed dramatically lower blood glucose levels. Particularly pre-treated nano-groups showed the greatest reduction in blood glucose levels (Fig. 4).

### Determination of biodistribution of NPIP in various tissues of experimental groups

The graphical illustration showed that mice administered with NPIP had higher concentrations of piperine in their liver, pancreas, spleen, kidney, and brain tissues than mice given PIP (Fig. 4).

### Analysis of % of viable cells in pancreatic tissue using Trypan Blue dye

Trypan Blue dye Exclusion test revealed the presence of fewer viable cells in the pancreas of the ALX-treated mice group compared to that of the untreated group used as a control. However, the ALX-induced pre-treated NPIP I + ALX group showed a greater number of live cells demonstrating NPIP I's ability to reduce ALX-induced pancreatic cell death more effectively (Fig. 4).

### Assessment of localization of NPIP in brain tissue of mice

Blue fluorescence was observed in the mice brain tissues that had been administered with both PIP and NPIP. Upon comparison of the fluorescence data of brain tissue of mice given PIP, with those given NPIP, the image from the NPIP-fed model showed a higher concentration of piperine. The experimental examination validated the capacity of NPIP to more effectively cross the BBB (Blood–Brain–Barrier) than PIP and additionally, supported PIP's capacity to cross the BBB, as predicted by the Egan BOILED–Egg study (Fig. 4).

### Assessment of chromosomal aberrations (CA) in experimental groups

**Figure 5:**
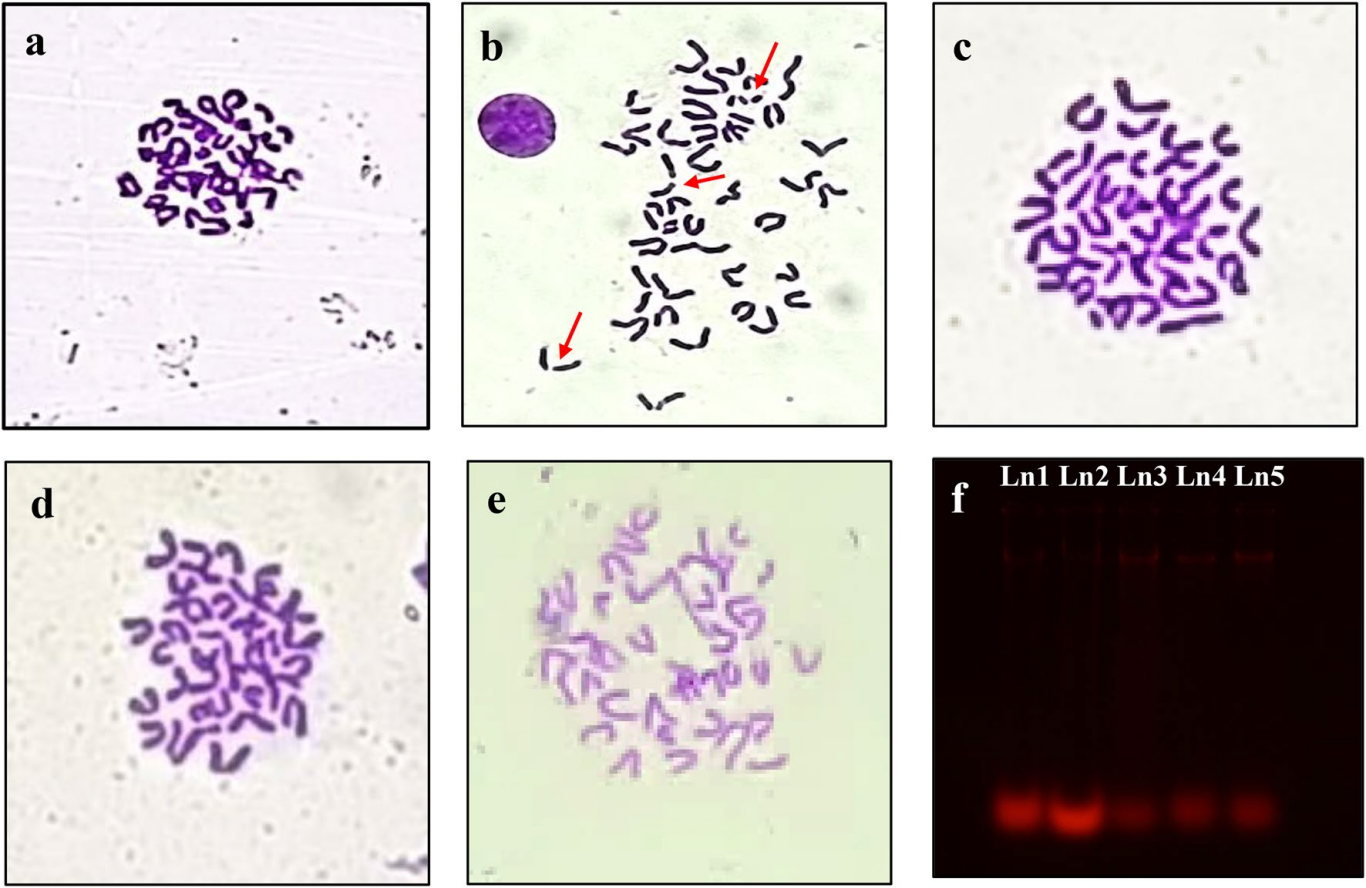
Evaluation of genotoxicity in mice model: Images showing the metaphasic chromosomal plates with arrows resembling aberrations (**a**: CONT; **b**: ALX; **c**: PIP + ALX; **d**: NPIP I + ALX; **e**: NPIP II + ALX) and DNA fragmentation study of pancreatic tissue of different experimental sets (**f**: Ln1-CONT GROUP; Ln2-ALX GROUP; Ln3-PIP + ALX; Ln4-NPIP I + ALX; Ln5-NPIP II + ALX) (Original gel are presented in Supplementary Fig. 3).

Alloxan administration resulted in numerous forms of chromosomal anomalies such as breakage of the chromosomal arm, ring/clustering of chromosomes, stretching, and so on. However, the incidence of CA was observed to be considerably lower in NPIP pre-treated + ALX sets when compared with ALX-treated sets, implying preventive effects of NPIP against ALX-induced CA, thereby maintaining the genomic integrity (Fig. 5a–e).

### DNA fragmentation study of pancreatic tissue

From the gel electrophoresis study, it became apparent that the ALX-treated set had a greater intensity of DNA smear demonstrating DNA fragmentation than the control sets. The fragmentation of DNA was significantly reduced in the pre-treated NPIP group following ALX administration. The figure additionally revealed the notable reduction in DNA damage observed in the group treated with NPIP II + ALX (Fig. 5f).

### Histological study of pancreatic tissue of experimental mice group

**Figure 6:**
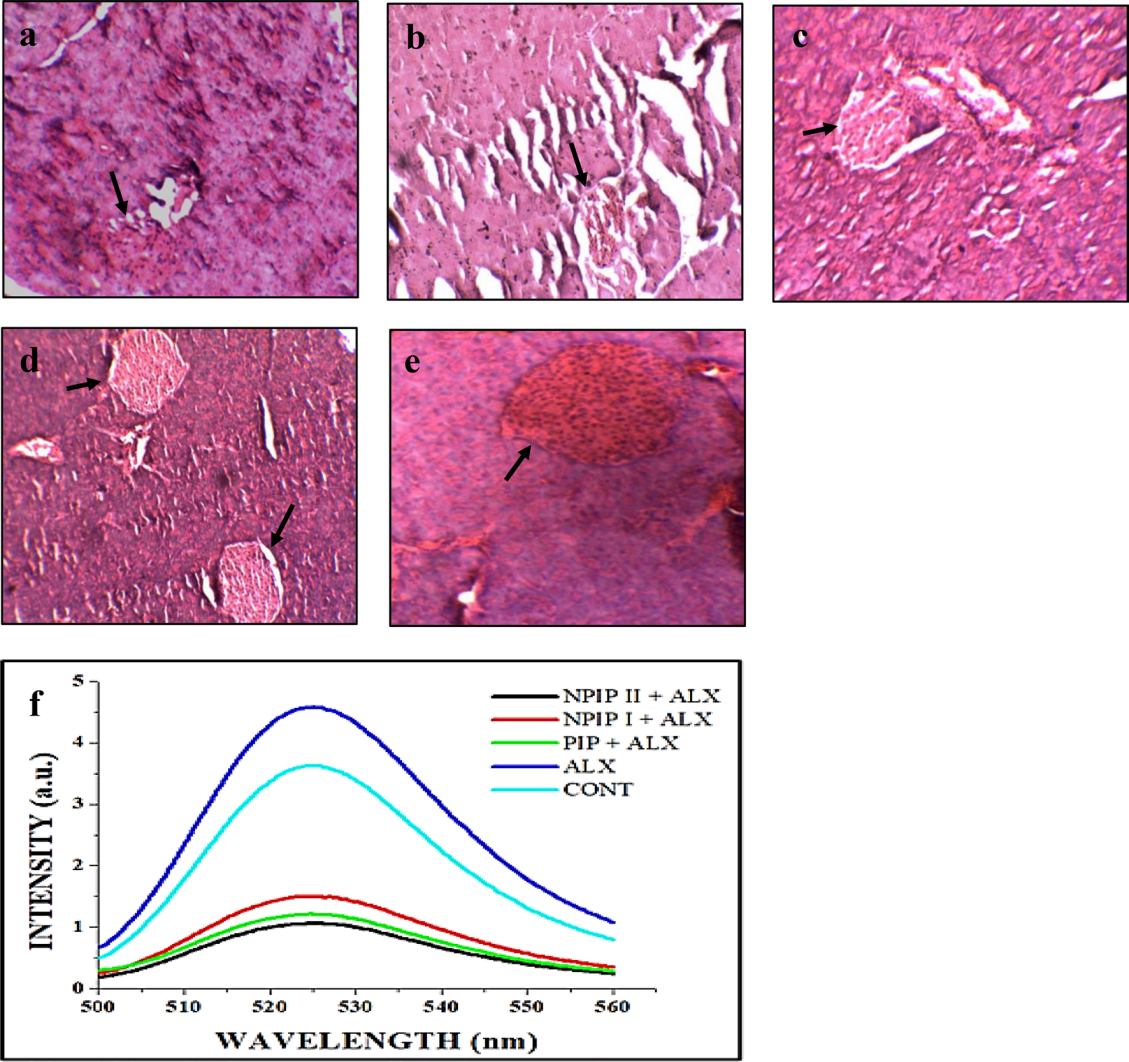
Histological analysis of pancreatic tissue (**a**: CONT; **b**: ALX; **c**: PIP + ALX; **d**: NPIP I + ALX; **e**: NPIP II + ALX) and Measurement of ROS: (**f**) Generation of ROS using H2DCFDA dye in pancreatic tissue of different experimental groups.

The histological study of pancreatic tissue from various experimental sets in the NPIP + ALX group showed a restoration of tissue structure that had been compromised after alloxan administration in the ALX group. In the pre-NPIP treated mice group, the islet cells and the acinar were more noticeable, providing evidence of the ability of the pancreas to restore insulin secretion in animals that had previously undergone NPIP treatment (Fig. 6a–e).

### Estimation of reactive oxygen species (ROS) production in pancreatic tissue

According to the spectrofluorimetric analysis, the ALX group showed a higher intensity of ROS production in the pancreatic cells in contrast to the untreated group and pre-treated NPIP group. Thus, the NPIP + ALX group could potentially circumvent oxidative stress by eliminating ROS (Fig. 6f).

### Immunofluorescence detection of PARP-1, p53, and Hsp90 protein expression

**Figure 7:**
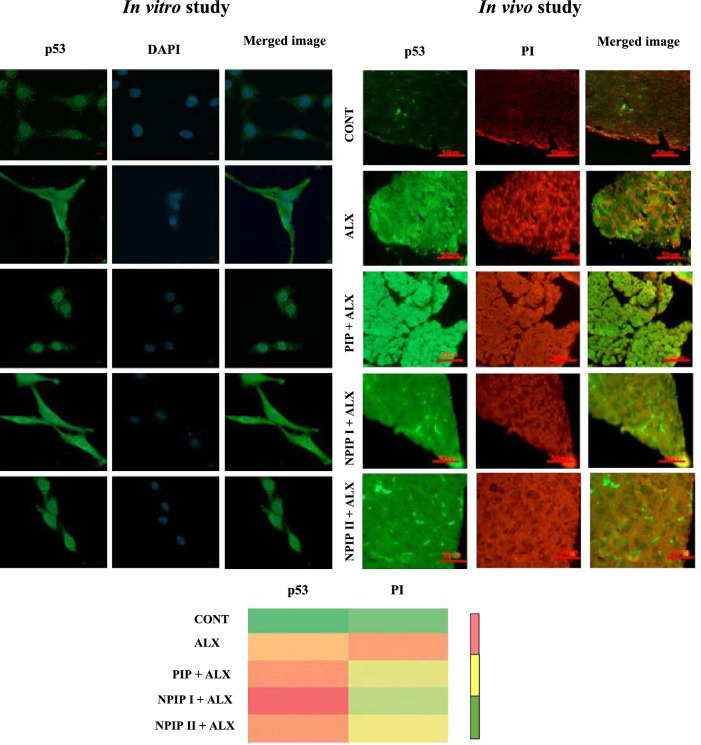
Immunofluorescence study: Expression level of p53 in in vitro L6 cell and in vivo pancreatic tissue with the study of nuclear condensation using DAPI and PI. Image of heatmap showing the intensity of p53 and PI in pancreatic tissue of mice (Pink colour: higher value, yellow colour: moderate value, green colour: lower value).

**Figure 8:**
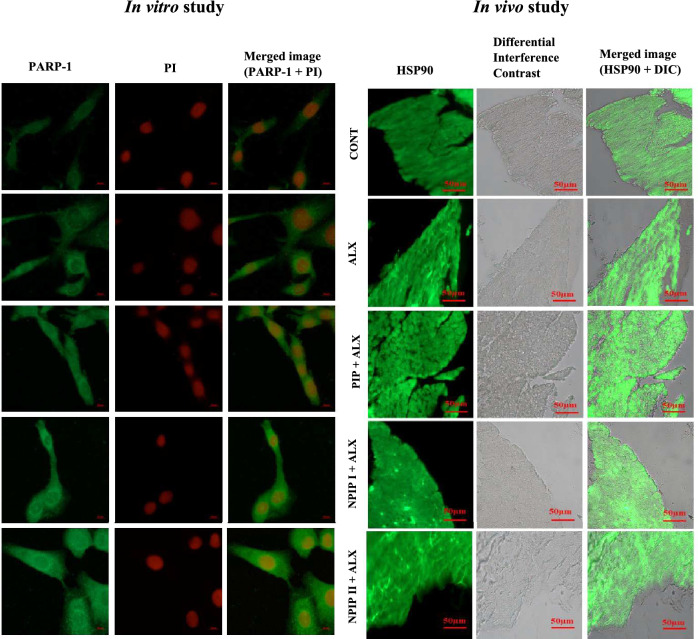
Immunofluorescence study of protein expression level: Immunofluorescence imaging of expression level of PARP-1 protein and fluorescence study of DNA damage using PI staining in vitro (left side) and HSP90 protein expression level observed by immunofluorescence study in pancreatic tissue in vivo (right side).

The results of the immunofluorescence study obtained from confocal microscopy images of pancreatic tissue demonstrated that the pre-treated NPIP mice group following ALX induction had a greater expression level of the p53, and Hsp90 proteins than the groups that only underwent ALX administration. The fluorescence images of the FITC-tagged secondary antibody followed by PI staining confirmed the lower incidence of DNA damage in the NPIP + ALX treated group and higher expression of p53 and Hsp90 protein. The ALX-treated group experienced greater nuclear condensation-based damage and decreased level of protein expression. The images of the in vitro study revealed that NPIP pre-treated cells showed higher intensity of PARP-1, p53, and Hsp90 protein expression than ALX-treated cells (Figs. 7, 8).

### Estimation of glucose uptake in experimental L6 cells (rat skeletal muscle cell line)

From the table, it was shown that cells preincubated with NPIP before administering ALX led to greater glucose uptake than PIP, indicating that it could have an integral aspect in alleviating hyperglycemic situations. Thus, NPIP might have an efficacious role in altering the ALX-induced reduction in glucose absorption in L6 cells (Suppl. Table 3).

### Detection of nuclear condensation in L6 cell line in vitro

To evaluate the effectiveness of NPIP in protection against ALX-induced nuclear condensation-based DNA damage, the intensities of DAPI (4′,6-diamidino-2-phenylindole) and PI (Propidium iodide) fluorescence were assessed, which revealed intact fluorescence in pre-treated NPIP group as compared to that in ALX (Figs. 7, 8).

## Discussion

The present work explored the anti-diabetic prospect of nano-encapsulated piperine based on the in silico prediction of targeting relevant proteins and ascertaining its druglikeness properties to combat the disease. The essence of nanotechnology lies in the fact that their nano size and negative zeta potential aid in their maximum cellular permeability and cellular uptake which leads to faster and greater action in a several-fold reduced dose than non-nano drugs. In this study, PIP is encapsulated within the biodegradable polymer PLGA to form the nanoparticles NPIP having a size of ∼ 30 nm (within the 100 nm range). Moreover, the threshold for the encapsulation or rather encapsulation efficiency of PIP in PLGA to form NPIP was 86.68% indicating that nanopiperine possesses a significant capacity to load piperine effectively. Thus, this ∼ 30 nm nanosized NPIP encapsulated in PLGA facilitated greater cellular absorption which was re-confirmed by computer-based Egan Boiled Egg model prediction. The lower molecular mass and negative zeta potential of NPIP could promote easier uptake, higher mobility, and stability into the tissue. Furthermore, the vital properties of PIP such as hydrogen bond donors, hydrogen bond acceptors, and XLogP value lie within the prescribed range of ADME parameters which in turn comprehensively establishes the biological suitability of the phytocompound. This helped in assessing the reason behind the experimental advantages of NPIP when administered in biological models encompassing easy penetration into lipid bilayers which had accelerated greater bio-distribution, biocompatibility, and bioavailability in pancreatic tissue to delineate the signalling cascades in combating diabetes in mice model and hyperglycemic stress in L6 cell line.

The generation of reactive oxygen species leads to a reduction in anti-oxidant enzyme production, which promotes disruption of insulin-secreting beta cells thereby, resulting in a rise in blood sugar levels as an incidence of diabetes. Administration of NPIP as pre-treatment inhibited oxidative tension by reducing the pool of reactive oxygen species formation and led to the restriction in ROS-induced cellular cytotoxicity. This result coincided well with the histological findings of compact pancreatic tissue affirming an elevated number of β cells in the pancreas which resulted in sustained insulin production for maintaining glucose homeostasis.

The therapeutic potential of a biologically active drug molecule depends on its efficacy in interacting with the active sites in DNA. The druggability of the compound increases with its ability to considerably bind to DNA and its likeliness to protect the DNA from damaging effects. NPIP when incubated with CT-DNA was found to interact well with the double-stranded DNA structure which ascertained the drug–nucleotide interaction when observed in CD spectra which would thereby bear testimony to the fact that the binding of NPIP to DNA initiates a change in the DNA structure that would aid in the protection of the DNA from being damaged by diabetes initiation and progression. This result was further supported by restriction in structural changes in chromosomes in NPIP pre-treated diabetic mice.

Redox imbalance had been implicated in the pathogenesis of diabetes which resulted in severe cellular oxidative stress-mediated impairment of islet cells. Since DNA and proteins are thought of as the genomic and functional building blocks of cells, safeguarding these macromolecular structures seemed imperative. The results of the immunofluorescence study provided evidence about the expression levels of p53, PARP-1, and Hsp90 in the pancreatic tissue of mice and L6 cells. Hsp90, a cytosolic protein, was the subject of the immunofluorescence investigation since it has been known to stabilize misfolded proteins. It has been reported that a deficit of insulin leads to protein catabolism. In the present findings, Hsp90 expression in the NPIP pre-treatment group appeared to exceed its expression level when compared to any other group. This increased expression of Hsp90 in the pre-drug treated group might infer the role of NPIP in preserving the protein integrity by stabilizing the structure through alkyl hydrophobic interactions with the residues of ALA55, LYS58, ILE96, LEU107, VAL150, MET98 and PHE138 which corroborated very well with our in silico molecular docked findings. ILE96, LEU107, VAL150, and ALA55 are hydrophobic residues having higher hydropathy indices. LYS58 is a hydrophilic residue. Therefore, it has been predicted that the presence of non-polar hydrophobic amino acid residues with higher hydropathy indices is favorable for the interactions with ligand components. Thus, the experimental findings and the docking result agreed that NPIP has an imperative role in protecting the protein from degradation. This protection from degradation after NPIP pre-treatment increases the expression of p53 and PARP-1 which in turn, facilitates the instigation of the DNA reparative cascades. Thus, a stable protein–ligand formation is ensured, which thereby negates hyperglycemia-induced damage and its underlined complications. PIP, the core component of NPIP, gets released from the PLGA capsule by biodegradation after being administered in mice or cell line, interacts strongly with PARP-1 and p53 proteins, resulting in its activation, enabling the initiation of a reparative pathway involving other replication machinery components and controlling DNA repair in the event of damage, thereby reducing overall DNA lesions. In the event of DNA repairing, the p53 was activated by PARP-1-mediated downstream repair signalling. Subsequently in the presence of ATP, the Hsp90 coupled with p53 induced the capacity to bind to the DNA promoter region with the correct folding conformation. By actively participating in PARP-1/p53 DNA repair signalling cascades, our formulated novel NPIP inhibited overall nuclear damage and genotoxicity triggered by diabetes/hyperglycemia, in both in vitro and in vivo model which might be dedicated to their greater tissue/ cellular internalization, penetration, and availability owing to their nano-size range when compared to unencapsulated/ naked PIP. Therefore, it can be stated that the NPIP may help in maintaining the integrity of Hsp90, which functioned as a fine-tuned mechanism to retain stable DNA repair proteins p53/PARP axis by shielding it against degradation from diabetes/hyperglycemia-induced oxidative stress-mediated genotoxicity and other complications.

Therefore, the concept of PLGA nano-drug delivery bridges the gap between the cost of drug procurement and dose dependency by controlling the quantity of the drug entering the body in a ten-fold reduced dose on one hand and acting in a target-specific manner on the other. Thus, our findings would pave a new alternative avenue in the arena of drug development for an optimized cost-effective approach to serve future diabetics with a better and longer life.

## Materials and methods

### Chemicals and reagents

Piperine, alloxan monohydrate, and many other chemicals utilized in the current research were of high analytical standards and were acquired from Sigma-Aldrich (St. Louis, MO, USA). Calf-thymus DNA (CT-DNA) and ethidium bromide were procured from SRL, India. The antibodies used were procured from Santa-Cruz Biotechnologies, USA. The company identities of various chemicals have been emphasized in the article based on their application in different studies.

### Formulation of PLGA-loaded nanopiperine (NPIP)

The formulation and preparation of nanopiperine (NPIP) was followed by its encapsulation in non-toxic, biodegradable polymer, poly-lactide-co-glycolide (PLGA) under optimal conditions^26–28^ using a conventional solvent displacement strategy.

### Characterization of NPIP using atomic force microscopy (AFM) and zeta potential

The surface characteristics, such as shape and size, of biosynthesized polymeric nano-based materials, were assessed using an AFM study following the standard technique by amplitude and tapping modes using the program WSxM 5.0 Develop 7.0^29–31^. The Zeta potential was measured using the Nano-ZS instrument, Malvern, UK following standard protocol^29,30^.

### Determination of %encapsulation efficiency (EE%) of NPIP

An absorption peak for piperine (PIP) was from 342 to 345 nm range which represented the λ_max_ of PIP. The drug entrapment efficiency/threshold for the encapsulation also denoted as % encapsulation efficiency (EE%) of PIP encapsulated within the polymer (PLGA) was calculated following a standard methodology. In brief, NANOSEP having a property of 100 kDa cut-off membrane filter was utilized for separating the unencapsulated i.e. unbound piperine from the PLGA-encased NPIP in a time bound manner^26^. Thereafter, the quantity of free piperine in the filtrate was calculated at the particular wavelength (absorption spectra of PIP) using a spectrophotometer (SHIMADZU UV-1700) and the %EE was calculated using the following equation:

$$\% Encapsulation\;Efficiency\;\left( {EE\% } \right) = \frac{{\left\{ {\left( {DRUG} \right)total - \left( {DRUG} \right)free} \right\}}}{{\left( {DRUG} \right)total}}\;*100$$

### CT-DNA binding study of PIP and NPIP using CD spectroscopy

The CD spectra interactions of PIP and NPIP with CT-DNA were measured within the 200–400 nm wavelength range following standard protocol^32^.

### In silico docking study

In silico docking is a highly improved software-based study that interprets receptor-ligand interaction by their complementarity at a molecular level^33^. Therefore, the preparation of both protein and ligand should be the prime step before docking. This study mainly focused on the following software: ChemDraw Ultra 8.0, Chem3D Ultra 8.0, Arguslab, and DiscoveryStudio. The ligand preparation began in ChemDraw Ultra 8.0 software (Fig. 3a) wherein the 2D structure of the ligand was drawn and converted into its 3D with the aid of Chem3D Ultra 8.0. Subsequently, the converted 3D figure was optimized for energy minimization by MM2 force field using a default Dielectric constant of 0.01 to gain equilibrium configuration of the respective molecules (Fig. 3b)^34^. The proteins of interest and their respective PDB IDs are PARP-1 (3GJW)^35^, p53 (1YC5)^36^, and Hsp90 (6LTI)^37^ and the co-crystallized ligands of the proteins in this study are 7-(pyrrolidin-1-ylmethyl)pyrrolo[1,2-a]quinoxalin-4(5H)-one, Nicotinamide and 5-[2,4-dihydroxy-5-(1-methylethyl)phenyl]-n-ethyl-4-[4-(morpholin- 4- yl-methyl)phenyl] isoxazole-3-carboxamide respectively. The target protein preparation was accomplished by downloading, and extracting the 3D form of protein from the Protein Data Bank (PDB). The final step of protein preparation by the addition of hydrogen atoms and the deletion of water molecules, was done in ArgusLab 4.0.1 software. The visualization of the interaction between the groups of ligands (drugs) with the amino acid residues of the receptor (protein) was done using the DiscoveryStudio.

### Druglikeness prediction of PIP

The phytocompound with good dock scores according to in silico simulation were further screened to evaluate their druglike attributes based upon Lipinski’s rule of 5 enabling it to be classified as a druglike molecule^38^.

### ADME analysis

An understanding of pharmacokinetics is necessary to assess and optimize the activity and effectiveness of bioactive chemicals. Following the consumption or administration of a drug, the compound's efficacy depends on its route inside the body^39^. This pathway of a drug is determined by its absorption, distribution, metabolism, and excretion which is termed ADME. Drug absorption is influenced by two variables, including human intestinal absorption (HIA) and membrane permeability based on Caco-2 Permeability. Drug distribution is governed by several factors, among which the blood–brain barrier (BBB) and distribution volume (VD) of a drug have been considered. The CYP (Cytochrome P450) models (CYP2D6, CYP3A4, CYP1A2, CYP2C19, CYP2C9) for substrate or inhibitor and total clearance model were used to predict metabolism and excretion of drug respectively^40^. The free web digital application, SwissADME, designed by the Swiss Institute of Bioinformatics, Switzerland^41^ and accessible at www.swissadme.ch, was used for in silico ADME analysis and screening of phytocompound. This phase of the screening technique was implemented for the molecules with high binding energy scores before any experimental studies.

### In vivo study

#### In vivo mice model

Male Swiss albino mice (*Mus musculus*) weighing approximately 20–25 g (b.w.) 6–8 weeks old were acclimatized for at least a week before conducting experiments by maintaining the temperature at 24–26 °C; humidity at 55 ± 5% and a 12-h light/dark cycle with free access to water and food. All the mice were housed in the above-mentioned identical conditions during the acclimatization and experimental period, and vital parameters like weight, and blood glucose level were monitored. A total of 30 mice were taken for conducting all the experiments according to the guidelines of the Animal Ethics Committee and the Animal Welfare Committee of the University of Kalyani following their approval (Institutional Animal Ethical Committee of University of Kalyani, 892/GO/Re/S/01/CPCSEA).

#### ARRIVE guidelines 2.0 statement

This research work followed the regulations of ARRIVE guidelines 2.0 which were related to this particular study.

#### Induction of diabetes in mice

Diabetes was developed in experimental mice by administering a single dose of intraperitoneal (IP) injection of 0.04% alloxan monohydrate (Sigma-Aldrich: CAS No. A7413) injected at a dosage of 100 mg/kg body weight^42^. The animals were provided access to a 5% glucose solution throughout the night followed by glucose monitoring observing standard practice^43^.

#### Administration of piperine and nanopiperine in mice

The mice were administered once daily with 40 mg/kg b.w. of the corresponding amount of PIP via oral gavage and fed for 14 consecutive days^44^. After the selection of piperine dose, an equivalent (40 mg/kg body weight) and reduced (20 mg/kg body weight) dosage of nanopiperine (NPIP) were administered to separate sets of mice.

#### Experimental design

The experimental sets were randomly chosen and split into five distinct groups. Each group contained six mice.

**Group I:** Untreated good healthy mice served as the control group; **Group II:** Alloxan (ALX) treated group served as the diabetic group; **Group III:** Animals were orally pre-administered with piperine (PIP) at a dose of 40 mg/kg body weight + alloxan induction; **Group IV:** Animals were orally pre-administered with nanopiperine (NPIP I) at a dose of 40 mg/kg body weight + alloxan induction; **Group V:** Animals were orally pre-administered with nanopiperine (NPIP II) at a dose of 20 mg/kg body weight + alloxan induction.

#### Measurement of glucose level in the blood of experimental mice group

Blood glucose levels were checked before and after the treatment period in all experimental sets, including the control group, and were tested following the standardized GOD-POD Glucose kit (Autospan) from ARKRAY Healthcare Pvt. Ltd., India (Code: 93DP100-74)^45,46^.

#### Bio-distribution study of PIP and NPIP in different tissues

The bio-distribution study was conducted by administering PIP and NPIP for 14 days to different groups of mice at a dosage of 40 mg/kg b.w. and 20 mg/kg b.w. respectively and analysed using a spectrophotometric measurement at the λ_max_ of the phytocompound (piperine), following standard practice^47^.

#### Cell viability study of pancreatic tissue using Trypan Blue dye

The in vivo cell viability test of isolated pancreatic tissues of experimental sets and the control group were assessed by Trypan Blue dye-exclusion method^10^ and the percentage cell viability was calculated using the following equation:

$$cell \;viability \left(\%\right)=\frac{Number \;of\; viable\; cells}{Total \;number \;of \;cells} *100$$

#### Assessment of the ability of NPIP to cross the blood–brain barrier (BBB)

To validate the software-based Egan Boiled egg method prediction, an experimental evaluation was made in an in vivo model by immunofluorescence study to determine the potential of NPIP and/or PIP to penetrate the BBB following common practice^48,49^.

#### Assessment of chromosomal aberrations in experimental groups

For a comprehensive analysis of chromosomal abnormalities between different experimental sets, chromosome metaphase spreads were prepared, appropriately stained using Giemsa and microscopically analysed for cytogenetical abnormalities following standard practice^50^.

#### Evaluation of in vivo DNA damage by fragmentation assay

The standardized phenol–chloroform technique was adopted to extract the genomic DNA from the pancreatic tissue of the different testing groups and agarose gel electrophoresis-based DNA-laddering or smearing was observed under UV transilluminator (GENEI)^51^.

#### Histological evaluation of pancreatic tissue

The histological sample preparation involves fixing, blocking and sectioning followed by Haematoxylin–Eosin (Haematoxylin: Merck, CAS No. 517-28-2; Eosin: Merck, Catalogue No. 109844) staining of tissues from autopsied mice, and analysing them under the compound light microscope.

#### Study of reactive oxygen species (ROS) generation using H_2_DCFDA staining

For detecting a cell's redox status directly, a Fluorescence Spectrophotometer (Hitachi F-7100) based determination of the fluorescence intensity of 2',7'-dichlorodihydrofluorescein diacetate (H_2_DCFDA) following its conversion to DCF was performed following common practice^52^.

### In vitro study

#### Cell culture

The L6 skeletal muscle cell line was procured from the National Centre for Cell Science (NCCS, Pune). The following conditions were used to sustain the cells: they were cultured in DMEM medium (Sigma) supplemented with 10% FBS (Gibco, Grand Island, NY, USA) and 1% penicillin–streptomycin (Sigma) and maintained in the incubator supplied with 5% CO_2_ at a temperature of 37 °C^30^. The in vitro study was conducted when the cells reached 70–75% confluency.

#### Preparation of alloxan

ALX has been selected for inducing hyperglycemia and incubated for 2 h at a concentration of 1 mM^26^.

#### Dose selection of PIP and NPIP

The cells were administered with different PIP treatment dosages as a pretreatment before being subjected to a normal dose of ALX for 2 h. For selecting the best PIP dosage for this experiment, the extent of glucose absorption was evaluated. 35 µg/100 µl of PIP was the dose that was used in our experiment. The dose of NPIP was chosen to be 35 µg/100 µl (higher dose) and 17.5 µg/100 µl (lower dose), respectively, based on this data.

#### Glucose concentration in the L6 cell line

The methods outlined in the standard GOD-POD kit technique^45^ were followed to determine the glucose concentration in L6 cells.

#### Analysis of nuclear condensation in L6 cells

In order to view the nuclear morphology in several experimental cell sets, propidium iodide (PI) and DAPI staining procedures were used on the cells and analysed appropriately following common practice^30^.

#### Immunofluorescence detection of p53, PARP-1, and Hsp90 protein expression levels in pancreatic tissue of mice and L6 cell line

Utilizing, anti-PARP-1, anti-p53, and anti-Hsp90 primary antibodies, as well as FITC (Fluorescein-5-isothiocyanate) labelled secondary antibodies, protein expression of PARP-1, p53, and Hsp90 in experimental and control groups were assessed using a confocal microscope (Carl Zeiss LSM 800)^30^.

### Statistical analysis

The data reported in our current study reflect the means of three separate trials. After computing the standard error of the mean values, a statistical evaluation of the data was done to assess the level of significance of differences between their mean values utilizing Student's t-test and analysis of variance (ANOVA).

### Supplementary Information

Supplementary Information.

## Data Availability

The data supporting the findings of the experiments are available from the corresponding author upon reasonable request.
