# Supplementary material for: Novel PLGA-encapsulated-nanopiperine promotes synergistic interaction of p53/PARP-1/Hsp90 axis to combat ALX-induced-hyperglycemia
Source: Sci Rep. 2024 Apr 25;14:9483. doi: 10.1038/s41598-024-60208-1 (PMC11045756; doi:10.1038/s41598-024-60208-1)
Supplement: Supplementary file 1 — Supplementary Information. [file 41598_2024_60208_MOESM1_ESM.pdf]

Supplementary file

A novel PLGA-encapsulated nanopiperine promotes the synergistic interaction of p53/PARP-1/Hsp90 axis to combat ALX-induced hyperglycemia

[1,2]Rishita Dey, [1]Sudatta Dey, [1]Priyanka Sow, [1]Arnob Chakrovorty, [1]Banani Bhattacharjee, [2]Sisir Nandi*, [1]Asmita Samadder*

[1]*Cytogenetics and Molecular Biology Laboratory, Department of Zoology, University of Kalyani, Kalyani, Nadia-741235, India.*

[2]*Department of Pharmaceutical Chemistry, Global Institute of Pharmaceutical Education and Research (Affiliated to Veer Madho Singh Bhandari Uttarakhand Technical University). Kashipur-244713, India*

**Corresponding authors: Dr. Asmita Samadder, Assistant Professor, Department of Zoology, University of Kalyani, Kalyani, Nadia-741235, India. E mail ID:* asmita.samadder@gmail.com, asmitazoo19@klyuniv.ac.in, *Phone: +91 9874548900 and Dr. Sisir Nandi, Professor and Head, Department of Pharmaceutical Chemistry, Global Institute of Pharmaceutical Education and Research (Affiliated to Veer Madho Singh Bhandari Uttarakhand Technical University) Kashipur-244713, India. E mail ID:* sisir.iicb@gmail.com, *Phone: +91 7500458478*

Materials and Methods

Assessment of % cellular toxicity

The % cellular toxicity was assessed in the pancreatic tissue of the experimental mice model (Swiss albino mice) prior to the experiment. Individual mice were given doses of piperine (PIP) and nanopiperine (NPIP) for a consecutive 14 days in order to evaluate the percentage of cellular toxicity. The mice were sacrificed following the 1st,2nd, 3rd,4th,…, 14th day to isolate the pancreas, and the toxicity of the drug was determined following the Trypan Blue dye method [Samadder et al., 2019].

Results

Determination of % cellular toxicity of PIP and NPIP

The % cellular toxicity was determined from Trypan Blue dye by assessing % cell viability after feeding PIP and NPIP to the mice group. The representative graph (Fig. 1) demonstrates that PIP and NPIP are considered safe and are not detrimental upon administration to a normal *in vivo* mouse model since even after being administered to the mice group, there was no significant cytotoxicity as the percentage of cellular viability was nearly identical to that of unfed mice (serving as a control).

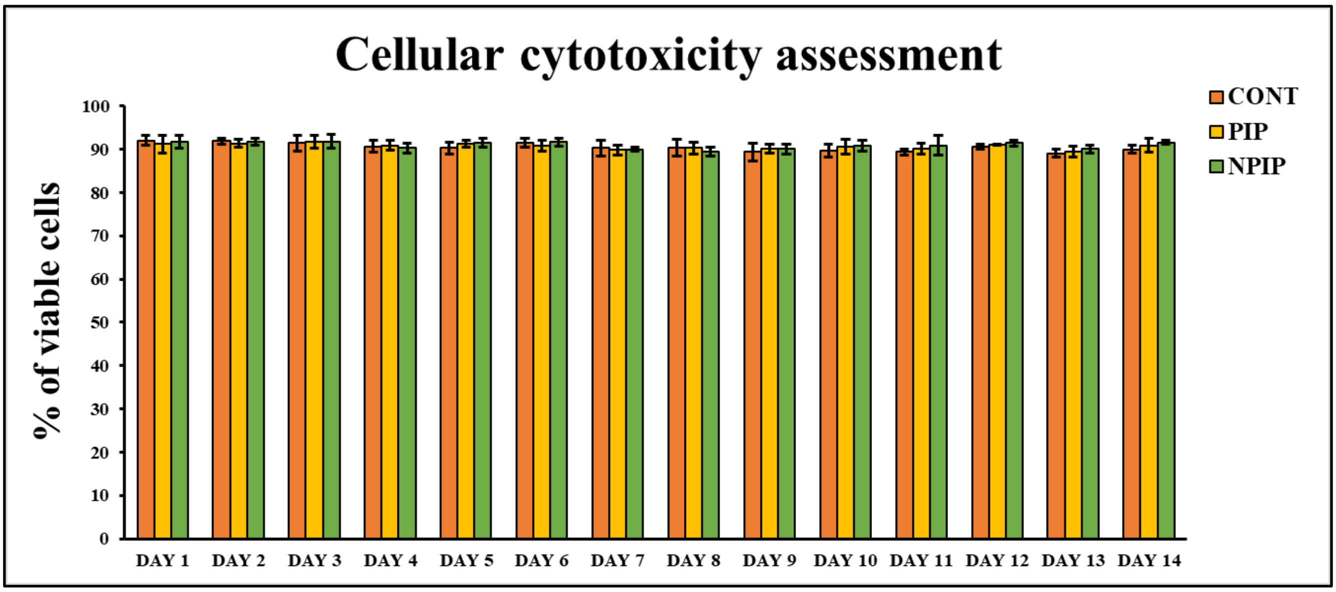

Fig 1: Cellular cytotoxicity assessment of PIP and NPIP

Assessment of %Encapsulation Efficiency (EE%) of PIP within NPIP

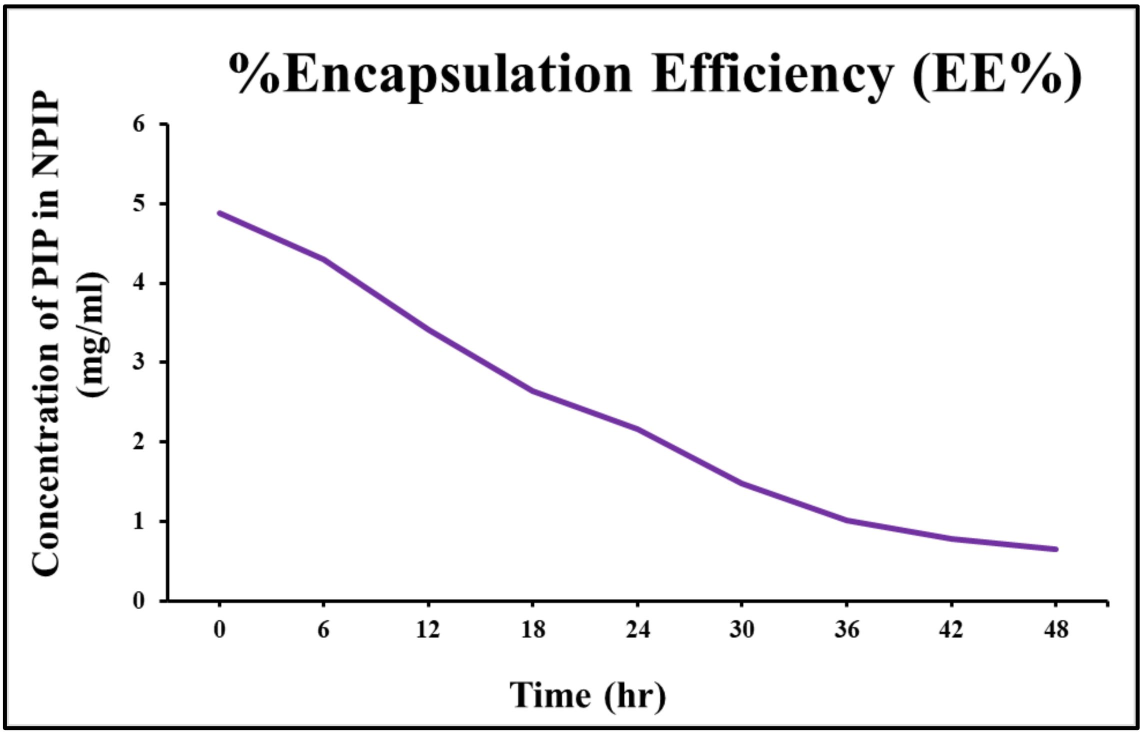

Fig 2: %Encapsulation Efficiency of PIP

DNA fragmentation study of pancreatic tissue

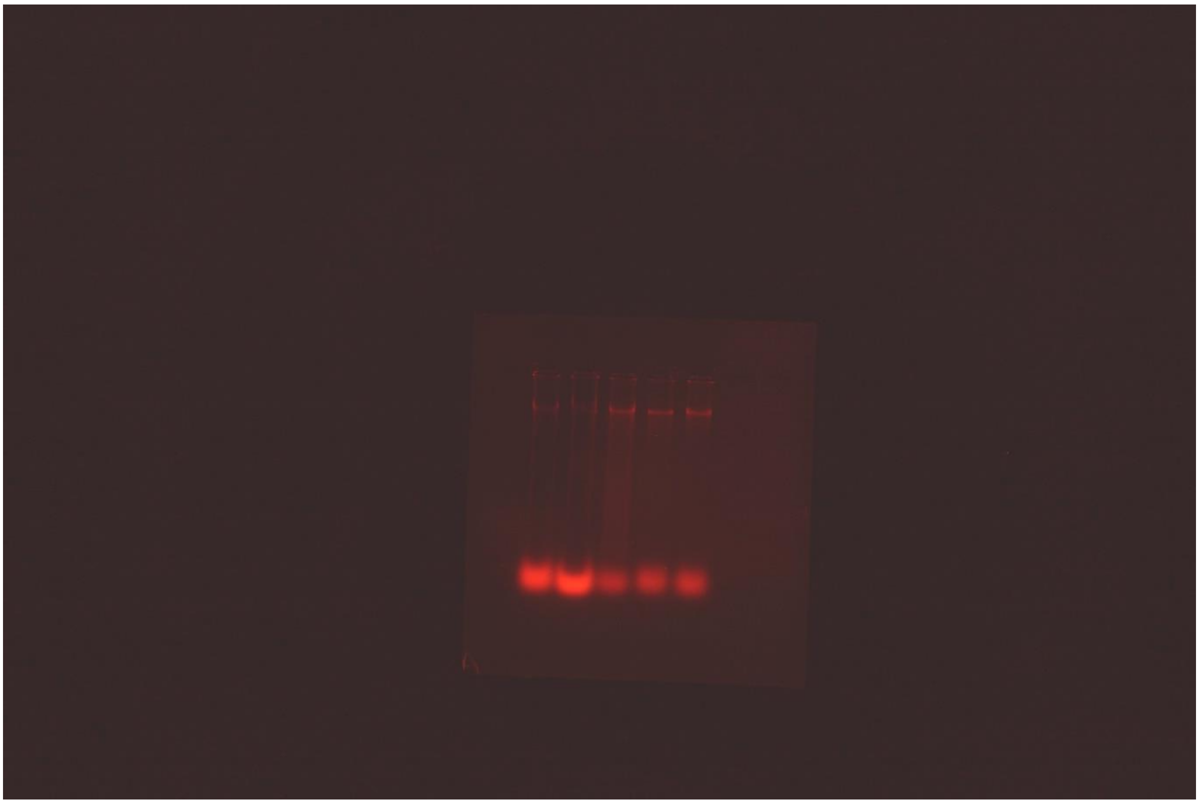

Fig 3a: DNA fragmentation study of pancreatic tissue of different experimental sets run in DNA GEL (f: Ln1- CONT GROUP; Ln2- ALX GROUP; Ln3- PIP + ALX; Ln4- NPIP I + ALX; Ln5- NPIP II + ALX)

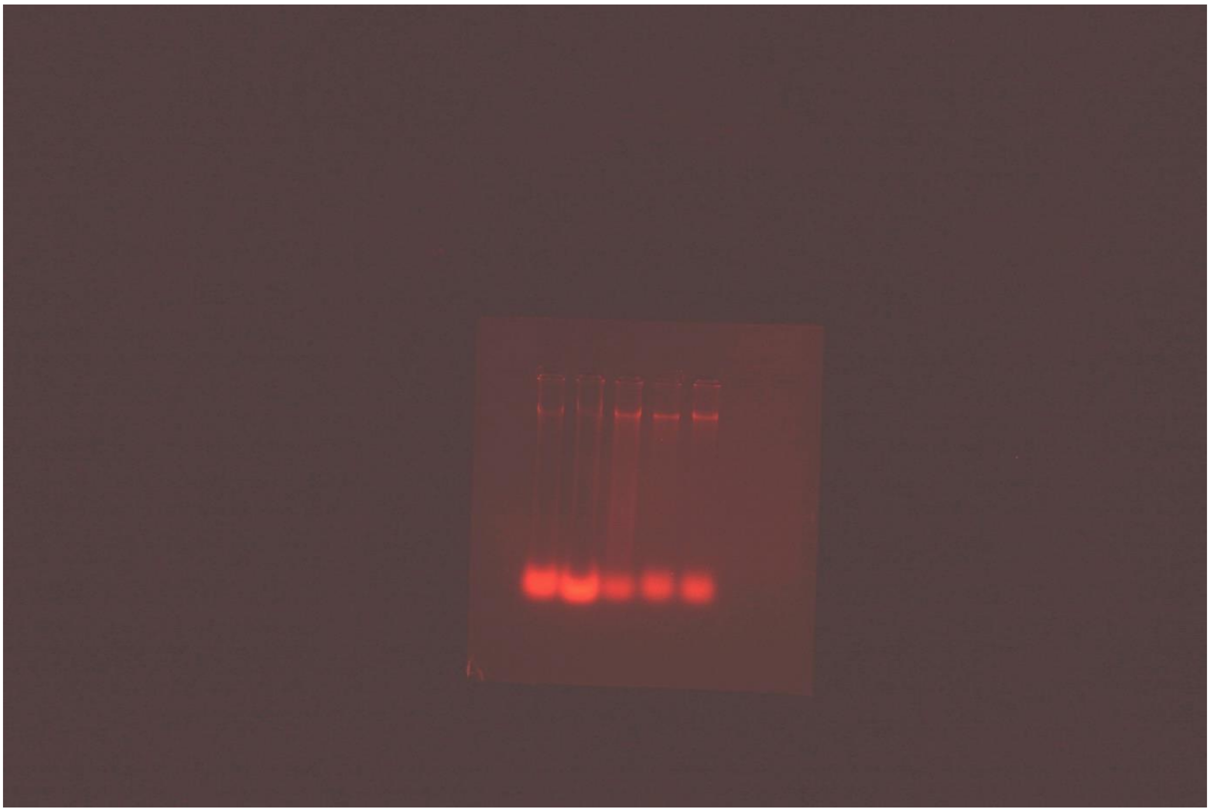

Fig 3b: DNA fragmentation study of pancreatic tissue of different experimental sets run in DNA GEL (f: Ln1- CONT GROUP; Ln2- ALX GROUP; Ln3- PIP + ALX; Ln4- NPIP I + ALX; Ln5- NPIP II + ALX) (Multiple/alternative exposure image)

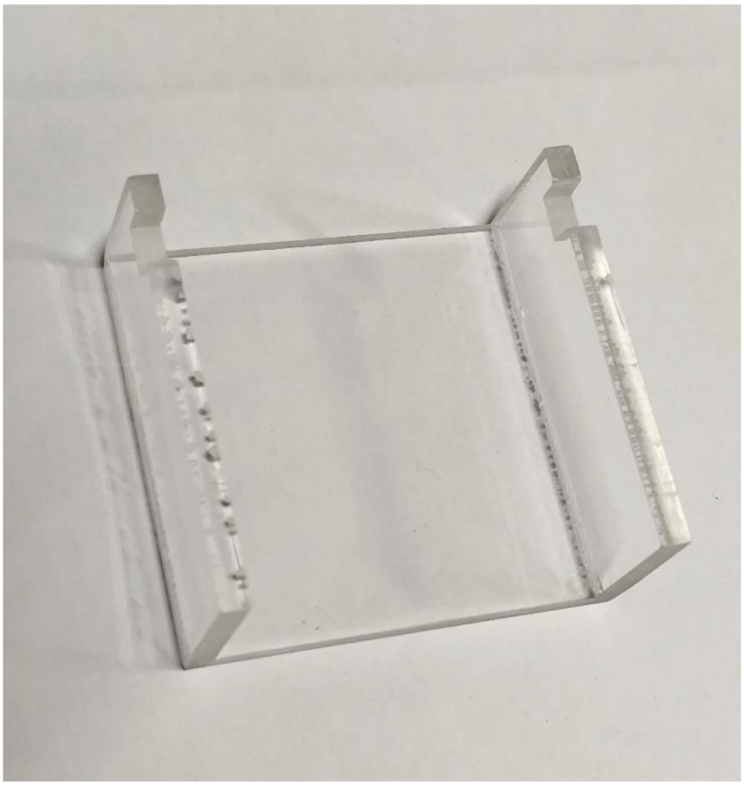

Figure 3c showing DNA GEL cast (where agarose is cast with EtBr for gel formation and subsequent DNA loading)

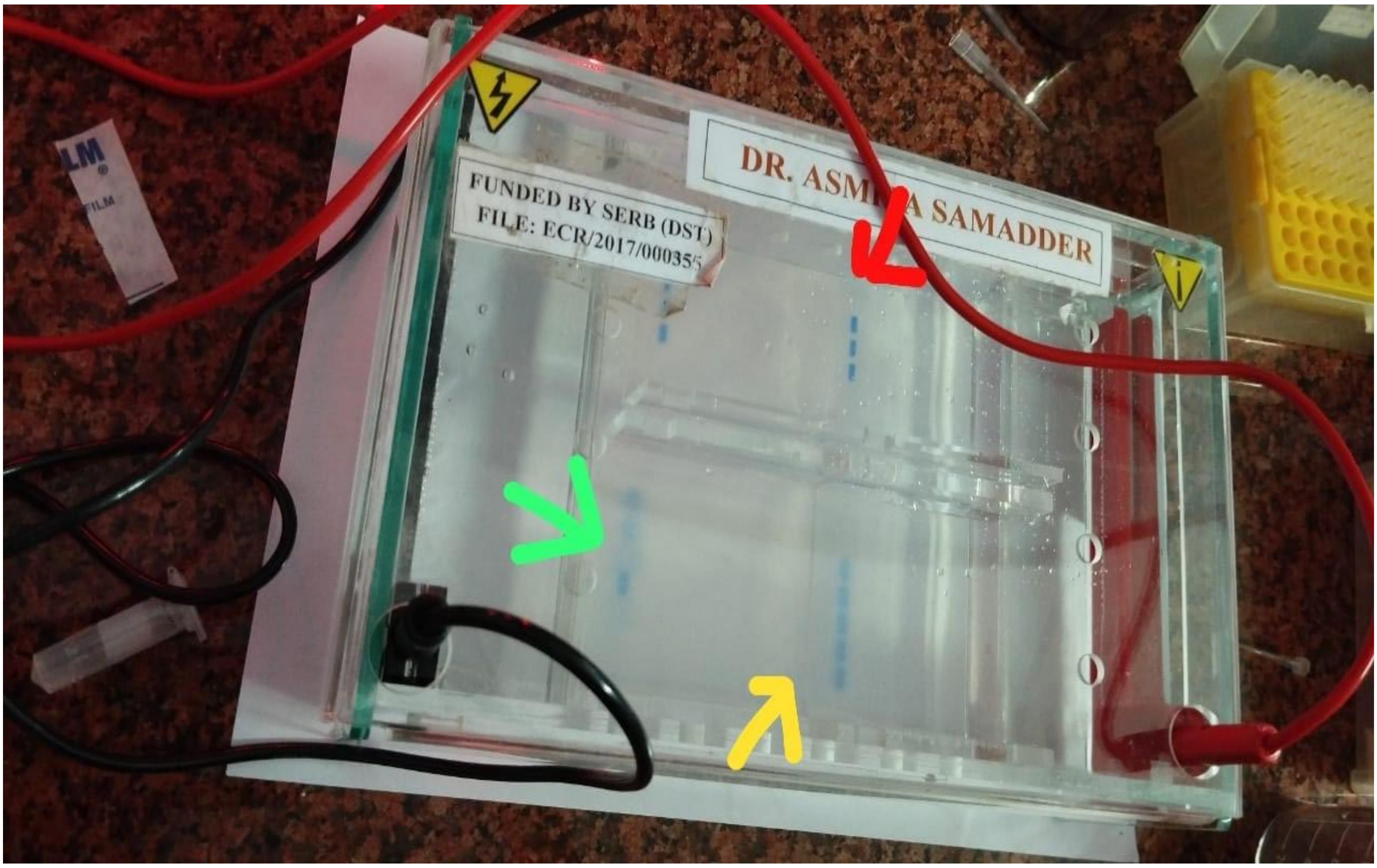

Figure 3d: DNA gel electrophoresis apparatus showing four separate agarose gel having 0.1% EtBr loaded with DNA running at the same time. The Yellow marked agarose gel cast loaded with DNA sample was run and after visualization under UV transilluminator was used as Figure 3a and 3b images for the DNA gel fragmentation assay.

Estimation of druglike property of PIP

Table 1: The Druglikeness properties of piperine

Molecular weight	285.34
Number of H-bond acceptors	3
Number of H-bond donors	0
Log Po/w (XLOGP3)	3.46

Estimation of ADME property of PIP

Table 2: Tabular representation of ADME property of PIP

ADME PROPERTIES	PARAMETERS	PREDICTION
**A- ABSORPTION**	Caco-2-Permeability	Yes
	Human intestinal absorption	Yes
**D- DISTRIBUTION**	Blood-Brain Barrier Penetration	Yes
**M- METABOLISM**	CYP2D6	Both inhibitor and substrate
	CYP34A	Inhibitor
**E- EXCRETION**	Clearance rate	Efficient clearance

Estimation of glucose uptake in experimental L6 cells (rat skeletal muscle cell line)

Table 3: Determination of the concentration of media glucose level in control and experimental L6 cells.

GROUP	MEDIA GLUCOSE LEVEL (mg/dl)
CONT	117.333±2.906
ALX	139.333±1.764 ##
PIP + ALX	115.000±2.646 **
NPIP I + ALX	109.333±1.202 ***
NPIP II + ALX	113.667±2.963 **

##p < 0.01 *vs* CONT, ****p* < 0.001 *vs* ALX, ***p* < 0.01 *vs* ALX were considered significant for Student's t-test

Reference

Samadder, A., Tarafdar, D., Das, R., Khuda-Bukhsh, A. R. &Abraham, S. K. Efficacy of nanoencapsulated pelargonidin in ameliorating pesticide toxicity in fish and L6 cells: Modulation of oxidative stress and signalling cascade. *Sci. Total Environ*. **671**, 466-73 (2019).
